# Comparing results of an exact vs. an approximate (Bayesian) measurement invariance test: a cross-country illustration with a scale to measure 19 human values

**DOI:** 10.3389/fpsyg.2014.00982

**Published:** 2014-09-08

**Authors:** Jan Cieciuch, Eldad Davidov, Peter Schmidt, René Algesheimer, Shalom H. Schwartz

**Affiliations:** ^1^University Research Priority Program ‘Social Networks,’ University of ZürichZürich, Switzerland; ^2^Institute of Psychology, Cardinal Stefan Wyszyński University in WarsawWarsaw, Poland; ^3^Institute of Sociology, University of ZürichZürich, Switzerland; ^4^International Laboratory for Socio-Cultural Research, National Research University-Higher School of EconomicsMoscow, Russia; ^5^Department of Political Science, University of GiessenGiessen, Germany; ^6^Department of Business Administration, University of ZürichZürich, Switzerland; ^7^Department of Psychology, The Hebrew University of JerusalemJerusalem, Israel

**Keywords:** multigroup confirmatory factor analysis, exact measurement invariance, approximate measurement invariance, configural metric scalar measurement invariance, revised Portrait Values Questionnaire, Bayesian analysis

## Abstract

One of the most frequently used procedures for measurement invariance testing is the multigroup confirmatory factor analysis (MGCFA). Muthén and Asparouhov recently proposed a new approach to test for approximate rather than exact measurement invariance using Bayesian MGCFA. Approximate measurement invariance permits small differences between parameters otherwise constrained to be equal in the classical exact approach. However, extant knowledge about how results of approximate measurement invariance tests compare to the results of the exact measurement invariance test is missing. We address this gap by comparing the results of exact and approximate cross-country measurement invariance tests of a revised scale to measure human values. Several studies that measured basic human values with the Portrait Values Questionnaire (PVQ) reported problems of measurement noninvariance (especially scalar noninvariance) across countries. Recently Schwartz et al. proposed a refined value theory and an instrument (PVQ-5X) to measure 19 more narrowly defined values. Cieciuch et al. tested its measurement invariance properties across eight countries and established exact scalar measurement invariance for 10 of the 19 values. The current study applied the approximate measurement invariance procedure on the same data and established approximate scalar measurement invariance even for all 19 values. Thus, the first conclusion is that the approximate approach provides more encouraging results for the usefulness of the scale for cross-cultural research, although this finding needs to be generalized and validated in future research using population data. The second conclusion is that the approximate measurement invariance is more likely than the exact approach to establish measurement invariance, although further simulation studies are needed to determine more precise recommendations about how large the permissible variance of the priors may be.

## Measurement invariance

Measurement invariance is a psychometric property of a scale developed to measure a latent construct. The instrument is measurement invariant when the same construct is measured in the same way across different groups, such as countries, cultural units, time points, or regions within countries (Horn and McArdle, 1992; Meredith, 1993; Vandenberg and Lance, 2000; Vandenberg, 2002; Millsap, 2011; Davidov et al., 2014). Measurement invariance is necessary for conducting meaningful comparisons across groups. The most widely used method to establish measurement invariance is multigroup confirmatory factor analysis (MGCFA; Jöreskog, 1971; Bollen, 1989). Usually one distinguishes between three levels of measurement invariance: configural (where all groups have the same pattern of factor loadings), metric (where the factor loadings are constrained to be equal across the compared groups), and scalar (where the factor loadings and the indicator intercepts are constrained to be equal across groups) (Vandenberg and Lance, 2000). Metric invariance is sufficient for comparing covariances and unstandardized regression coefficients across groups. A meaningful comparison of latent means across groups, however, requires the scalar level of measurement invariance.

Some researchers have argued that partial (metric or scalar) measurement invariance is sufficient for meaningful comparisons (Byrne et al., 1989; Steenkamp and Baumgartner, 1998). Partial invariance is supported when the parameters of at least two indicators (loadings at the metric level and loadings plus intercepts at the scalar level of the measurement) are equal across groups.

Measurement invariance is becoming an increasingly important and disputed topic in the social sciences. To illustrate, in April 2014, the term “measurement invariance” yielded about 239,000 hits in a Google Scholar search. This abundance of scientific papers falls into three categories. The first category includes methodological papers that introduce, discuss, and evaluate various methods and approaches to measurement invariance. The second includes papers that test the measurement invariance of a given construct across groups as a precondition for further comparative analysis. These papers assess measurement invariance as a preliminary analysis that allows for a meaningful test of the substantive hypotheses. The third category of papers reports the measurement invariance properties of specific questionnaires that were developed to measure specific latent constructs. These papers assess the quality of the questionnaires for analyses within and across countries or time points. They seek to improve questionnaire validity and reliability by identifying weaknesses and problems in the formulation of questions, in translation, in culture appropriateness, and so on. Establishing measurement invariance in one study does not signify that a questionnaire is always measurement invariant. Measurement invariance should be repeatedly tested across groups, because noninvariance can be caused by external features of the study in addition to internal features of the instrument.

The aim of the present study is two-fold. First, we try to establish the measurement invariance properties of Schwartz et al.'s (2012) newly developed scale to measure human values. This goal locates the present study in the third category of studies listed above. Second, we apply two methods (exact and approximate) for establishing measurement invariance and compare their findings. This goal locates the present study in the first category of studies listed above. The approximate approach for testing measurement invariance is more liberal than the exact approach. However, extant knowledge about how results of approximate measurement invariance tests compare to the results of the exact measurement invariance test is missing. We address this gap by comparing the results of exact and approximate (Bayesian) cross-country measurement invariance tests of the revised scale to measure human values. We query whether the approximate (more liberal) approach yields higher levels of measurement invariance for the values scale than the exact approach.

## Schwartz's theory of basic human values

Schwartz (1992), Schwartz et al. (2012) defines values as broad, trans-situational goals that vary in importance and serve as guiding principles in the life of a person or group. Schwartz distinguishes between value hierarchies and value structure. Value hierarchies refer to the relative importance of the set of values to different individuals. The central claim of Schwartz's value theory concerns the value structure. It asserts that values form a circular motivational continuum. This means that values that are located in adjacent regions on the continuum are motivationally similar. Behavior that expresses one value is likely to express the adjacent values at the same time. In contrast, values that are located on opposing sides of the circle express conflicting motivations; hence, behavior that expresses one value is likely to simultaneously challenge or block the expression of opposing values in the circle.

**Figure 1:**
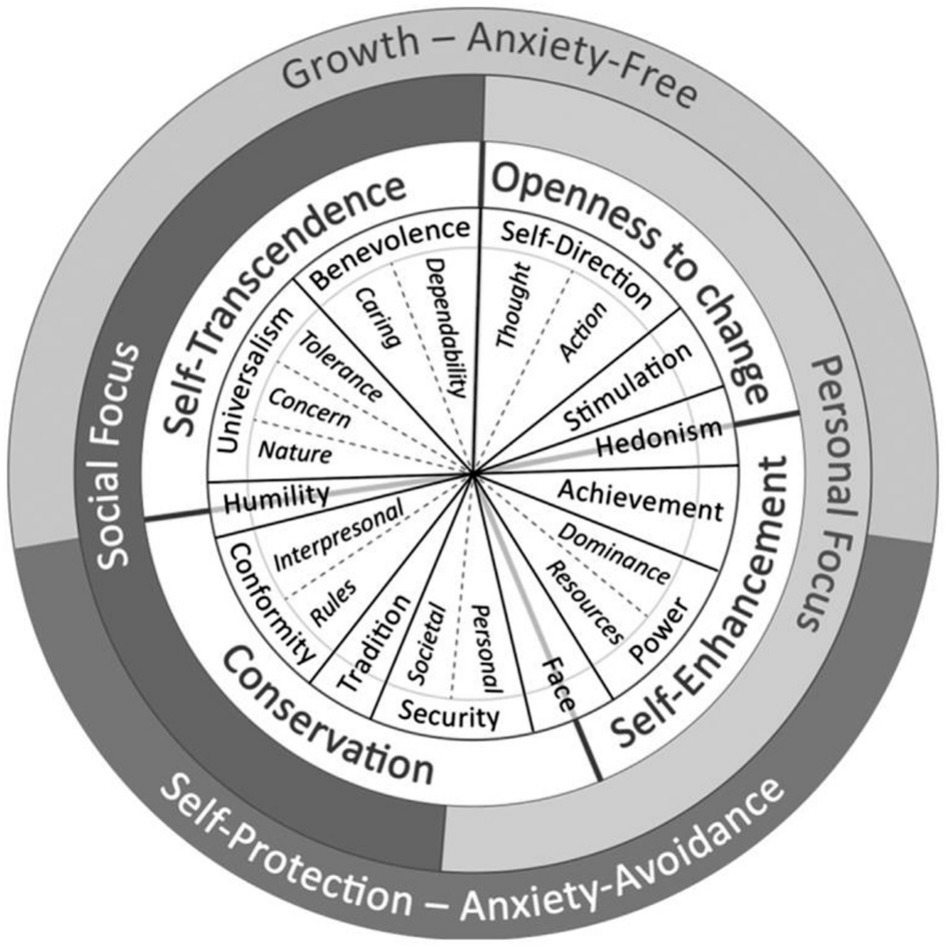
**The circular motivational continuum of 19 values in the refined value theory (Cieciuch et al., 2014)**.

The claim that values form a continuum implies that the circle of values can be partitioned in any number of ways. Depending on the aims of a study, one can differentiate between fewer broadly defined values or many more narrowly defined values. There are two common ways of partitioning the circular continuum, the classic version and the refined version. The classic version (Schwartz, 1992) partitions the circle into 10 basic human values. The refined version (Schwartz et al., 2012) partitions the circle into 19 more narrowly defined values. The 19 values in the refined version are subdimensions of the 10 basic human values (Schwartz et al., 2012). The values in both versions can be grouped into sets of four higher-order values: person-oriented vs. socially-oriented values or self-protection vs. growth values. Thus, the refined version of the theory and the classic version both describe the same circular motivational continuum. However, the refined theory provides a more discriminate partitioning of the continuum, thus allowing more fine-tuned predictions and explanations. Figure 1 depicts the value circle with its 19 narrowly defined values, and the definition of each value is presented in Table 1.

**Table 1:** **Nineteen more narrowly defined values in the refined theory of values (Schwartz et al., 2012)**.

**Value**	**Conceptual definitions in terms of motivational goals**
Self-direction—Thought	Freedom to cultivate one's own ideas and abilities
Self-direction—Action	Freedom to determine one's own actions
Stimulation	Excitement, novelty, and change
Hedonism	Pleasure and sensuous gratification
Achievement	Success according to social standards
Power—Dominance	Power through exercising control over people
Power—Resources	Power through control of material and social resources
Face	Security and power through maintaining one's public image and avoiding humiliation
Security—Personal	Safety in one's immediate environment
Security—Societal	Safety and stability in the wider society
Tradition	Maintaining and preserving cultural, family, or religious traditions
Conformity—Rules	Compliance with rules, laws, and formal obligations
Conformity—Interpersonal	Avoidance of upsetting or harming other people
Humility	Recognizing one's insignificance in the larger scheme of things
Benevolence—Dependability	Being a reliable and trustworthy member of the ingroup
Benevolence—Caring	Devotion to the welfare of ingroup members
Universalism—Concern	Commitment to equality, justice, and protection for all people
Universalism—Nature	Preservation of the natural environment
Universalism—Tolerance	Acceptance and understanding of those who are different from oneself

## Measurement of basic human values

The problem of measurement invariance is especially important for values because researchers often use them to describe differences between demographic, occupational, cultural, and national groups (Inglehart and Baker, 2000; Schwartz, 2006). Several methods have been developed to measure the values in Schwartz's approach. Currently, the most commonly used questionnaires are several versions of the Portrait Value Questionnaire (PVQ). The original version (PVQ-40) includes 40 items (Schwartz et al., 2001; Schwartz, 2003). A shorter version, implemented in the European Social Survey (ESS), includes 21 items (PVQ-21, Schwartz, 2003). The most recent version, developed to measure the 19 values of the refined value theory, includes 57 items (PVQ-57, Schwartz et al., 2012).

Several studies have tested the measurement invariance across countries of the PVQ-21 with data collected in the ESS (e.g., Davidov, 2008, 2010; Davidov et al., 2008). These studies succeeded in identifying only seven values at the configural level; it was necessary to unify some pairs of adjacent values in the confirmatory factor analyses. Davidov et al. (2008) established metric invariance for these seven values, but not scalar invariance. The lack of scalar invariance even for these seven was problematic because it meant that comparisons of means across cultures or countries may not be meaningful.

Cieciuch and Davidov (2012) addressed this problem when they compared the invariance properties between the PVQ-21 and PVQ-40 across Poland and Germany. They found that the PVQ-40 displayed a higher level of measurement invariance than the PVQ-21; it attained scalar invariance for all of the values except stimulation. They attributed the superiority of the PVQ-40 to the larger number of indicators available to measure the latent factors. With more items, the possibility of establishing partial scalar invariance increases. The reason for this is that, when establishing partial invariance, researchers need to identify at least two items with equal parameters across groups. When the number of indicators measuring a construct increases, chances also increase to identify two such items.

To measure all of the narrowly defined values that are differentiated in the refined theory, Schwartz et al. (2012) developed the PVQ-57. This version introduced three important changes compared to previous versions of the PVQ: (1) Single sentences were used for all items, replacing the two-sentence items of earlier versions. This avoided the dangers associated with double-barreled questions and improved overall clarity. (2) All items referred to the “importance” of a valued goal or characteristic to the respondent, replacing terms that referred to desires and feelings in earlier versions. This increase in consistency ensured that all items fit the conception of values as goals that vary in importance. (3) Three items measured each of the 19 values, which is in contrast to the varying number of items for each value in the PVQ-40 and the two items in the PVQ-21.

CFA analyses of the revised PVQ instrument successfully identified all 19 values in eight countries (Finland, Germany, Israel, Italy, New Zealand, Poland, Portugal, and Switzerland), establishing both configural and metric invariance (Cieciuch et al., 2014). Moreover, Cieciuch et al. (2014) established scalar measurement invariance for items measuring 10 of the 19 values across the eight countries. **Table 5** presents the detailed results of these analyses. Encouraging as these findings are in allowing comparison of means across countries for 10 values, a problem remains with the other nine values for which scalar invariance was not established. Perhaps, however, the method used to test measurement invariance test was overly strict. We therefore asked whether a more liberal test would yield more invariant results.

## The current study

Several researchers have recently argued that, although measurement invariance is necessary for meaningful comparisons across groups, the criteria for evaluating measurement invariance are too strict (Muthén and Asparouhov, 2013; Van de Schoot et al., 2013; Muthén, 2014). This may lead to rejecting the possibility of comparison and needlessly discourage research in some cases. Adopting this view, Muthén and Asparouhov (2013) proposed the concept of approximate rather than exact measurement invariance, which is based on Bayesian analysis.

### Approximate (Bayesian) measurement invariance

Bayesian analysis allows researchers to introduce existing knowledge into their analyses, especially the amount of uncertainty. The current practice within the dominant frequentist approach is to use existing knowledge in the theoretical introduction of papers and in the discussion but seldom in the analyses. Often the testing of null hypotheses ignores the existence of prior knowledge. Bayesian analysis allows testing informative hypotheses, that is, hypotheses that take prior knowledge into account. This logic may also be applied to testing measurement invariance.

In the Bayesian approach, parameters (e.g., loadings or intercepts) are considered to be variables with a specific distribution. The parameters of this distribution are called priors and can be defined by the researcher based on previous knowledge or assumptions (Muthén and Asparouhov, 2013). In the exact measurement invariance approach, researchers assume that the differences between loadings (or intercepts) across groups are zero or, in other words, that the loadings (or intercepts) are exactly equal across groups. The Bayesian measurement invariance approach introduces the concept of approximate equality. Thus, for testing approximate measurement invariance, one can expect that some differences in loadings (or intercepts) can occur, however, the mean of the differences between loadings (or intercepts) across groups is zero. Because the low variability is rather random, a normal distribution of the differences in loadings (or intercepts) with zero mean and small variance is assumed. Several simulation studies have shown that small variations (variance equal to 0.01 or 0.05) in the distribution of the differences in loadings or intercepts do not bias substantive conclusions for comparative research (Muthén and Asparouhov, 2013; Van de Schoot et al., 2013). Consequently, it makes sense to regard a small amount of variation as acceptable. Approximate measurement invariance differs from the partial measurement invariance approach, because in the latter some parameters are constrained to be exactly equal and others are released entirely, while in the former all parameters are constrained; however, the restrictions are more liberal and refer to the concept of approximate equality.

In the next section we test for approximate measurement invariance of the 19 values from the refined value theory of Schwartz et al. (2012). We then compare the findings to those established in previous studies that used exact measurement invariance testing.

Approximate measurement invariance is a relatively new approach. Therefore, there are few comparisons in the literature of the results that this approach yields with those obtained by the classic, exact measurement, invariance approach. We expect that the new scale to measure 19 values will exhibit a higher invariance level than the one reported by Cieciuch et al. (2014) when approximate measurement invariance is applied, because it allows for small differences between parameters that are otherwise constrained to be exactly equal in the exact measurement invariance approach. This would justify doing additional cross-cultural comparisons.

## Methods

### Participants and procedure

We used the same data employed for testing exact measurement invariance in Cieciuch et al. (2014). Data were from the following countries: Finland (*N* = 334, 65% female, *M*_age_ = 42.3, *SD*_age_ = 6.1), Germany (*N* = 325, 77% female, *M*_age_ = 23.4, *SD*_age_ = 5.0), Israel (*N* = 394, 65% female, *M*_age_ = 25.7, *SD*_age_ = 6.2), Italy (*N* = 388, 59% female, *M*_age_ = 35.6, *SD*_age_ = 14.5), New Zealand (*N* = 527, 68% female, *M*_age_ = 19.5, *SD*_age_ = 4.2), Poland (*N* = 547, 66% female, *M*_age_ = 27.0, *SD*_age_ = 10.0), Portugal (*N* = 295, 58% female, *M*_age_ = 27.0, *SD*_age_ = 10.4), and Switzerland (*N* = 201, 70% female, *M*_age_ = 28.8, *SD*_age_ = 7.7). All participants were contacted by researchers or instructed assistants in person or online and completed the value instrument voluntarily and anonymously. Data were collected in a written format in Finland, Germany, Italy, Poland, and in half the Portuguese sample. Data were collected online in the remaining samples. All data are available from the first author upon request.

### Questionnaire

Data were collected with the PVQ-5X (Schwartz et al., 2012) developed to measure 19 more narrowly defined values. Items described a person in terms of what is important for him or her (gender matched). The respondents were asked to answer the question “*How much is this person like you*” on a scale ranging from 1 (*not like me at all*) to 6 (*very much like me*). For example, the question “Freedom to choose what he does is important to him” measured the self-direction value. The question “Obeying all the laws is important to her” was used to measure the value conformity rules. All items are presented in **Table 4**. We excluded nine items which did not load satisfactorily on their corresponding value in the study of Schwartz et al. (2012). Thus, our analyses included exactly the same items included in the exact measurement invariance test of Cieciuch et al. (2014). Ten of the values were measured by three indicators and nine values by two indicators. Missing values for all items were below 0.7% with the exception of one achievement item (AC1) which had 2.9% missing values.

## Analysis

### Testing for approximate measurement invariance in Mplus (version 7.11)

The approximate measurement invariance test procedure is included in Mplus (Muthén and Muthén, 1998–2012) in the mixture analysis framework. Mixture modeling means that besides the latent variables included in the model, there are also one or more latent categorical variables that describe membership of respondents to a certain class. These latent categorical variables represent homogenous subpopulations of the studied heterogeneous population (Muthén, 2002). In principle, mixture modeling assumes that the division into subpopulations and subpopulation membership are not known but can be inferred from the data. However, in our case this was a straightforward inference, because the population membership was deduced by the country where data on the individuals were collected. Thus, this categorical variable was known, since it was simply the variable that described membership in groups (countries). In terms of mixture models, this situation is known as a single-class mixture model because there is only one class (one categorical variable). According to Asparouhov and Muthén (2010), if the categorical variable is observed, the single-class mixture model is essentially the same as a multigroup model. Kim et al. (2013) also argue that the two models (i.e., the multigroup model and the single-class mixture model with known class membership) are in principle the same.

Table 2 presents the syntax, briefly explains the various steps of the analysis, and provides a description of the statements used in the syntax.

**Table 2:** **Mplus syntax for approximate measurement invariance test and explanations (this is an example for a single factor—UNC)**.

**VARIABLE:**	
Names are country UNC1 UNC2 UNC3;	This indicates the variables in the data: the countries and the items for each value (Universalism-concern in this example).
classes = c(8);	This option specifies that there is one latent categorical variable (named c) that has 8 latent classes. The number 8 refers to 8 countries in the analysis.
knownclass = c(country = 1 2 3 4 5 6 7 8);	This option defines the categorical latent variable by the observed variable. There are 8 classes and respondents with value 1 in variable “country” belong to the first one; respondents with value 2 in variable “country” belongs to the second country, etc. If all values from the variable are to be analyzed, the statement can be shortened: knownclass = c (country).
**ANALYSIS:**	
type = mixture;	Approximate measurement invariance is included in Mplus within the mixture modeling analysis framework. The number of classes is known because it corresponds to the number of groups to be compared.
Estimator = bayes;	Bayesian analysis will be performed and priors can be defined.
chains is 5;	The number of chains in Markov chain Monte Carlo (MCMC) algorithms. The default in Mplus is 2 chains and the researcher can increase the number of chains by this statement.
Processor = 5;	To increase the speed of computation, one can use more processors if they are available in the hardware. It is possible to specify the number of processors that is equal to number of chains. In this case one can specify also 8 processors. If that many processors are not available, each available processor carries out one chain and after it is completed starts with the next chain.
Biterations = 500,000(20,000);	This option is used to specify the maximum and minimum number of iterations for each Markov chain Monte Carlo algorithm. In this case, it specifies that a minimum of 20,000 and a maximum of 50,000 iterations will be used.
Bconvergence = 0.01;	Specification of the convergence value criterion to be used for determining convergence of the Bayesian estimation.
bseed 100;	Specification of the seed to be used for a random number generation in the Markov chain Monte Carlo (MCMC) algorithm (the default in Mplus is zero).
model = allfree;	Factor means, variances, and covariances are freely estimated across groups with the exception of factor means in the last group which are fixed to 0.
**MODEL:**	
%overall% UNC by UNC1* UNC 2 UNC 3 (lam#_1-lam#_3); [UNC 1 UNC 2 UNC 3] (nu#_1-nu#_3);	In the mixture models, the label “%overall%” introduces the model description which is common for all groups. In this case the latent variable is loaded by three indicators (UNC1, UNC2, and UNC3). The asterisk after UNC1 implies that the loadings of the first indicator, which is usually constrained by default to 1, is freed.
	Following the “by” statement, the names of the factor loadings are listed in parentheses. One row below, after the brackets, the names of the intercepts are listed. It is necessary to list these so that one can later define their priors.
**MODEL PRIORS:**	
do(1,3) diff(lam1_#-lam8_#)~N(0,0.01); do(1,3) diff(nu1_#-nu8_#)~N(0,0.01);	The statement defines priors for loadings and intercepts. The distribution of loadings and intercepts is normal with mean = 0 and variance = 0.01
%c#8% [UNC @0]; UNC @1;	The label “%c#8%” refers to the part of the model for class 8 that differs from the overall model. In this case, the latent mean of UNC in the last group is constrained to 0 and the variance to 1 in order to identify the model according to the proposal of Muthén and Asparouhov (2013).

### Evaluation of the model

The fit of the Bayesian model can detect whether actual deviations are larger than those that the researcher allows in the prior distribution. The model fit can be evaluated based on the posterior predictive probability (ppp) value and the confidence interval (CI) for the difference between the observed and replicated chi-square values. According to Muthén and Asparouhov (2013) and Van de Schoot et al. (2013), the Bayesian model fits the data when the ppp is higher than zero^1^ and the CI contains zero. We defined the mean of the differences in loadings and intercepts across countries as zero and the variance of these differences as 0.01 (Van de Schoot et al., 2013). If the model was unacceptable based on the ppp and the CI, we slightly increased the variance to determine the level of variation in the priors for the difference between loadings and intercepts that would lead to acceptable model fit coefficients^2^. Additionally, Mplus lists all parameters that significantly differ from the priors. This feature is equivalent to modification indices in the exact measurement invariance approach. While the model is assessed based on ppp and CI, these values provide global model fit criteria that are similar to the criteria in the exact approach (Chen, 2007). Although several parameters have been identified as exactly equal in Cieciuch et al. (2014), we did not constrain them to equality and allowed a wiggle for the differences between all factor loadings and intercepts. The reason is that we wanted to assess whether a liberal model would establish invariance for all values.

## Results

Table 3 presents the fit coefficients of the approximate multigroup CFA for each value separately. For most of the values, the ppp was not significant, and the 95% CI for the difference between the observed and replicated chi-square values contained zero, which means that the approximate scalar invariance models for these values are acceptable. The only three exceptions were stimulation, achievement, and humility. Therefore, we increased the variance prior for these values to 0.02. With this adjustment, all three approximate scalar invariance models were also acceptable for these values. In other words, the model fit criteria suggest that approximate invariance could be established for all 19 values across eight countries.

**Table 3:** **Model fit coefficients of Bayesian multigroup confirmatory factor analysis for each value**.

	**ppp**	**95% *CI***
Self-direction–-Thought	0.201	(−19.478) – (49.818)
Self-direction–-Action	0.112	(−12.931) – (57.474)
Stimulation	0.001	(25.824) – (110.628)
Stimulation, prior of variance = 0.02	0.081	(−9.495) – (64.259)
Hedonism	0.258	(−18.255) – (35.833)
Achievement	0.004	(20.132) – (98.707)
Achievement, prior of variance = 0.02	0.103	(−13.481) – (62.092)
Power–-Resources	0.367	(−22.056) – (30.480)
Power–-Dominance	0.208	(−15.653) – (37.917)
Face^*^	0.128	(−11.916) – (45.275)
Security–-Personal	0.361	(−20.384) – (32.179)
Security–-Societal	0.135	(−13.923) – (55.015)
Tradition	0.028	(−0.594) – (76.570)
Conformity–-Rules	0.352	(−20.444) – (30.633)
Conformity–-Interpersonal	0.083	(−11.226) – (65.544)
Humility^*^	0.009	(6.575) – (70.861)
Humility, prior of variance = 0.02	0.121	(−11.877) – (46.340)
Benevolence–-Caring	0.506	(−34.843) – (33.737)
Benevolence–-Dependability^*^	0.149	(−12.476) – (43.798)
Universalism–-Concern	0.235	(−25.179) – (47.297)
Universalism–-Nature	0.167	(−18.021) – (51.002)
Universalism–-Tolerance	0.395	(−23.183) – (31.304)

ppp = posterior predictive p-value; 95% CI = Confidence interval for the difference between the observed and the replicated chi-square values,

* because of estimation problems, the latent means were constrained to 0 and variances to 1 in two countries for this value rather than in one country. These additional constraints were not rejected by the model.

Several loadings and intercepts in various countries deviated from the defined priors. For example, the intercept of the first item measuring Self-direction–Thought (SDT1) deviated from the defined prior in two countries, Finland and Poland. The loading of the first item measuring Stimulation (ST1) deviated in two countries, Italy and Poland, and its intercept deviated from the defined prior in two countries as well, Italy and New Zealand. Table 4 presents all deviations of loadings and intercepts from the defined priors. Despite the deviations listed in Table 4, the ppp and CI reached acceptable levels, which suggests that approximate metric and scalar measurement invariance are supported by the data for all values.

**Table 4:** **Deviations of loadings and intercepts from prior defined parameters (mean = 0, variance = 0.01)**.

	**Finland**		**Israel**		**Italy**		**New Zealand**		**Poland**		**Portugal**		**Switzerland**		**Germany**	
	**Lo**	**Int**	**Lo**	**Int**	**Lo**	**Int**	**Lo**	**Int**	**Lo**	**Int**	**Lo**	**Int**	**Lo**	**Int**	**Lo**	**Int**
SDT1 Being creative is important to him		x								x						
SDT2 It is important to him to form his own opinions and have original ideas						x										
SDT3 Learning things for himself and improving his abilities is important to him		x				x				x		x				
SDA1 It is important to him to make his own decisions about his life	x	x				x		x								
SDA2 Doing everything independently is important to him		x				x		x		x					x	
SDA3 Freedom to choose what he does is important to him					x	x										
ST1 He is always looking for different kinds of things to do					x	x		x	x							
ST2 Excitement in life is important to him			x						x			x		x		x
ST3 He thinks it is important to have all sorts of new experiences		x				x						x	x	x		
HE1 Having a good time is important to him						x			x	x						
HE2 Enjoying life's pleasures is important to him																
AC1 He thinks it is important to be ambitious				x					x	x		x				x
AC2 Being very successful is important to him																
AC3 He wants people to admire his achievements				x						x		x				x
POR1 Having the feeling of power that money can bring is important to him																
POR2 Being wealthy is important to him																
POD1 He wants people to do what he says						x										
POD3 It is important to him to be the one who tells others what to do						x										
FAC1 It is important to him that no one should ever shame him										x						
FAC2 Protecting his public image is important to him										x						
SEP2 His personal security is extremely important to him																
SEP3 It is important to him to live in secure surroundings																
SES1 It is important to him that his country protect itself against all threats																
SES2 He wants the state to be strong so it can defend its citizens										x						
SES3 Having order and stability in society is important to him		x	x	x					x	x						
TR1 It is important to him to maintain traditional values or beliefs		x		x		x		x		x				x		x
TR2 Following his family's customs or the customs of a religion is important to him		x								x				x		x
TR3 He strongly values the traditional practices of his culture												x				
COR2 It is important to him to follow rules even when no one is watching								x								
COR3 Obeying all the laws is important to him																
COI1 It is important to him to avoid upsetting other people		x			x	x		x	x	x		x				
COI2 He thinks it is important never to be annoying to anyone				x		x		x		x						
COI3 He always tries to be tactful and avoid irritating people				x		x										x
HU2 It is important to him to be humble																
HU3 It is important to him to be satisfied with what he has and not to ask for more																
BEC1 It's very important to him to help the people dear to him															x	
BEC2 Caring for the well-being of people he is close to is important to him												x			x	
BEC3 (BED1) it is important to him to be loyal to those who are close to him					x											
BED2 He goes out of his way to be a dependable and trustworthy friend							x	x								
BED3 He wants those he spends time with to be able to rely on him completely	x			x					x	x			x	x		x
UNC1 Protecting society's weak and vulnerable members is important to him								x								x
UNC2 He thinks it is important that every person in the world have equal opportunities in life																
UNC3 He wants everyone to be treated justly, even people he doesn't know								x				x			x	x
UNN1 He strongly believes that he should care for nature		x		x		x		x							x	
UNN2 It is important to him to work against threats to the world of nature		x										x				
UNN3 Protecting the natural environment from destruction or pollution is important to him								x								
UNT2 It is important to him to listen to people who are different from him																
UNT3 Even when he disagrees with people, it is important to him to understand them																

Lo = loading; Int = intercept; x—deviation of a given parameter in a given group from the defined priors (mean = 0, variance = 0.01).

Table 5 presents a comparison of Cieciuch et al.'s (2014) results using the exact approach and the results in the current study obtained using the approximate approach. Whereas exact scalar invariance was previously supported only for a subset of the 19 values, in the present analysis, approximate measurement invariance was established for all values, including those values where exact measurement invariance testing failed to display scalar invariance. In the next section we are going to discuss in more detail the results, their implications, and limitations.

**Table 5:** **Comparison of exact and approximate measurement invariance of 19 values across eight countries**.

	**Exact (Cieciuch et al., 2014)**		**Approximate (the current study)**
	**Metric**	**Scalar**	**Metric and scalar**
Self-direction thought	Full in all countries	Partial in all countries	Full in all countries
Self-direction action	Full in five countries, partial in Finland and Portugal, absent in Italy	Full in all countries	Full in all countries
Stimulation	Full in all countries	Full in all countries	Full in all countries^*^
Hedonism	Full in seven countries, Absent in Switzerland	Full in six countries, absent in Switzerland, Poland	Full in all countries
Achievement	Full in six countries, partial in Finland and Poland	Absent in all countries	Full in all countries^*^
Power dominance	Full in all countries	Full in six countries, absent in Portugal, Italy	Full in all countries
Power resources	Full in all countries	Full in seven countries, absent in Poland	Full in all countries
Face	Full in all countries	Absent in all countries	Full in all countries
Security personal	Full in all countries	Full in six countries, absent in Israel and Switzerland	Full in all countries
Societal security	Full in seven countries, partial in Portugal	Partial in all countries	Full in all countries
Tradition	Full in all countries	Absent in all countries	Full in all countries
Conformity rules	Full in all countries	Absent in all countries	Full in all countries
Conformity interpersonal	Full in all countries	Absent in all countries	Full in all countries
Humility	Full in all countries	Absent in all countries	Full in all countries^*^
Universalism nature	Full in all countries	Full in four countries, partial in Israel, Italy, and New Zealand, absent in Switzerland	Full in all countries
Universalism concern	Full in all countries	Full in five countries, partial in New Zealand, Portugal, absent in Germany	Full in all countries
Universalism tolerance	Full in all countries	Full in six countries, absent in Poland and Portugal	Full in all countries
Benevolence caring	Full in all countries	Full in seven countries, partial in Finland	Full in all countries
Benevolence dependability	Full in all countries	Absent in all countries	Full in all countries

* The allowed variance for the cross-country difference between intercepts and the loadings was 0.02. In all other cases it was 0.01.

## Summary and conclusions

Measurement invariance is a precondition for meaningful cross-group comparisons. Assuming rather than empirically testing whether the precondition is satisfied can be dangerous and can lead to wrong conclusions. Therefore, an empirical test of measurement invariance of a study's measures is necessary. However, the classic (exact) test is very demanding and very often leads to the rejection of measurement invariance and to precluding group comparisons. Van de Schoot et al. (2013) metaphorically described this situation as traveling between Scylla and Charybdis. Scylla represents the situation in which a model lacks measurement invariance, whereas Charybdis represents the situation in which the model was not tested for measurement invariance. In both situations, the researcher cannot know whether the differences between groups are real and substantive or a result of methodological artifacts. We followed Van de Schoot et al. (2013) suggestion to choose a third option for traveling between Scylla and Charybdis. This option is the approximate Bayesian approach to measurement invariance. Approximate measurement invariance is a rather new approach and applications using it and comparing its findings to those of the exact approach are rare. Using data on human values in eight countries, we tried to fill this gap by comparing the findings of an earlier analysis using the exact approach to measurement invariance by analyzing the same data using the approximate approach.

The approximate approach established measurement invariance across eight countries for the new PVQ-5X scale to measure human values even in cases in which the exact approach did not. In other words, the approximate method is less restrictive than the exact, and our findings suggest that—as expected—the results align with this, i.e., the less restrictive method (approximate invariance testing using the Bayesian procedure) produces stronger invariance than the exact approach did. These findings provide, for the first time, initial encouraging results that the PVQ-5X scale may be used for conducting meaningful cross-cultural research with all 19 values. The exact approach to assessing invariance has often shed doubt on the invariance of many questionnaires. The current findings provide hope that empirical testing for measurement invariance in questionnaires is not necessarily doomed to failure. Researchers may now put their scales to even a stricter test and examine whether some of the parameters may be constrained to be exactly (rather than approximately) equal.

Findings raise the question whether other established scales to measure human values such as the PVQ-21 scale included in the ESS will display higher levels of equivalence across countries when using the approximate Bayesian (rather than an exact) approach for the test. Future research should address this question by investigating the cross-country comparability of other scales to measure human values using the Bayesian approximate invariance approach.

This study is not without limitations. First, we used convenience student samples and data were collected using different modes of data collection (online and offline). Although previous studies (e.g., Davidov and Depner, 2011) demonstrated that online and offline modes of data collection produce invariant value measurements, future studies should address this issue by trying to validate and generalize our findings using country population samples. Second, we do not know whether and to what extent the different sample sizes across countries (e.g., 547 in Poland vs. 201 in Switzerland) may have disproportionally biased the fit measures. In his simulations, Chen (2007) provided recommendations for model fit evaluation for different sample sizes when testing for exact measurement invariance. However, we are not aware of any such simulations for the Bayesian approach. Future research should address the robustness of the model fit criteria to different sample sizes. Furthermore, it is not clear whether and to what extent the fact that the outcomes are ordinal might affect the results. Whereas exact measurement invariance tests can take the ordinal character of item scores into account in the estimation, unfortunately, the Bayesian approach does not deal with this problem appropriately and assumes that scores are continuous. We can only speculate that this may bias our conclusions but it is difficult to judge in which direction. Future research should address this problem by developing Bayesian procedures that allow testing for approximate measurement invariance while taking into account the ordinal character of the data. Yet it should be noted that our response scale included six categories, one more than the common five-point Likert scales, so this should have hopefully mitigated the problem.

In spite of our encouraging findings, an important unanswered question remains to be resolved: What is the magnitude of the variance that should be specified for the priors? Specifying a small variance may result in failure to establish invariance while specifying a larger variance may lead to establishing invariance. We set a magnitude of 0.01 and in three cases increased it to 0.02 in order to establish invariance. These seem like small magnitudes, but are they too liberal? This technical question is extremely important from an applied point of view. Finally, it is too early to claim that researchers should now switch to testing for approximate measurement invariance (instead of testing for exact measurement invariance). It is still a rather unexplored field, and further studies are needed before such a claim can be fully justified. In addition to the promising results reported here, further research and simulation studies should focus on these questions to provide guidelines for applied researchers.

### Conflict of interest statement

The authors declare that the research was conducted in the absence of any commercial or financial relationships that could be construed as a potential conflict of interest.

## Footnotes

^1^Simulation studies are still required to determine what level of probability researchers may rely on.

^2^There are still no established cut-off criteria in the literature about the maximal level of variability that may be used for the priors.

## References

[B1] Asparouhov T. Muthén B. O. (2010). Bayesian Analysis Using Mplus: Technical Implementation. Available online at: http://www.statmodel.com/download/Bayes3.pdf

[B2] Bollen K. A. (1989). Structural Equations with Latent Variables. New York, NY: Wiley

[B3] Byrne B. M. Shavelson R. J. Muthén B. O. (1989). Testing for the equivalence of factor covariance and mean structures–the issue of partial measurement invariance. Psychol. Bull. 105, 456–466 10.1037/0033-2909.105.3.456

[B4] Chen F. F. (2007). Sensitivity of goodness of fit indexes to lack of measurement invariance. Struct. Equ. Modeling 14, 464–504 10.1080/10705510701301834

[B5] Cieciuch J. Davidov E. (2012). A comparison of the invariance properties of the PVQ-40 and the PVQ-21 to measure human values across German and Polish samples. Surv. Res. Method 6, 37–48

[B6] Cieciuch J. Davidov E. Vecchione M. Beierlein C. Schwartz S. H. (2014). The cross-national invariance properties of a new scale to measure 19 basic human values. A test across eight countries. J. Cross Cult. Psychol. 45, 764–779 10.1177/0022022114527348

[B9] Davidov E. (2008). A cross-country and cross-time comparison of the human values measurements with the second round of the European Social Survey. Surv. Res. Method 2, 33–46

[B10] Davidov E. (2010). Testing for comparability of human values across countries and time with the third round of the European Social Survey. Int. J. Comp. Sociol. 51, 171–191 10.1177/0020715210363534

[B11] Davidov E. Depner F. (2011). Testing for measurement equivalence of human values across online and paper-and-pencil surveys. Qual. Quant. 45, 375–390 10.1007/s11135-009-9297-9

[B12] Davidov E. Meuleman B. Cieciuch J. Schmidt P. Billiet J. (2014). Measurement equivalence in cross-national research. Annu. Rev. Sociol. 40, 55–75 10.1146/annurev-soc-071913-043137

[B13] Davidov E. Schmidt P. Schwartz S. (2008). Bringing values back in. The adequacy of the European Social Survey to measure values in 20 countries. Public Opin. Q. 72, 420–445 10.1093/poq/nfn035

[B14] Horn J. L. McArdle J. J. (1992). A practical and theoretical guide to measurement invariance in aging research. Exp. Aging Res. 18, 117–144 10.1080/03610739208253916 1459160

[B15] Inglehart R. Baker W. E. (2000). Modernization, cultural change, and the persistence of traditional values. Am. Sociol. Rev. 65, 19–51 10.2307/2657288

[B16] Jöreskog K. G. (1971). Simultaneous factor analysis in several populations. Psychometrika 36, 409–426 10.1007/BF02291366

[B17] Kim S. Y. Mun E. Y. Smith S. (2013). Using mixture models with known class membership to address incomplete covariance structures in multiple-group growth models. Br. J. Math. Stat. Psychol. 67, 94–116 10.1111/bmsp.12008 23432382 PMC3864537

[B18] Meredith W. (1993). Measurement invariance, factor-analysis and factorial invariance. Psychometrika 58, 525–543 10.1007/BF02294825

[B19] Millsap R. E. (2011). Statistical Approaches to Measurement Invariance. New York, NY: Routledge

[B20] Muthén B. O. (2002). Beyond SEM: general latent variable modeling. Behaviormetrika 29, 81–117 10.2333/bhmk.29.81

[B21] Muthén B. O. (2014). IRT studies of many groups: the alignment method. Front. Psychol. 5:978 10.3389/fpsyg.2014.00978 PMC4162377 25309470

[B22] Muthén B. O. Asparouhov T. (2013). BSEM Measurement Invariance Analysis. Mplus Web Notes: No. 17. Available online at: www.statmodel.com

[B23] Muthén L. Muthén B. O. (1998–2012). Mplus User's Guide, 7th Edn. Los Angeles, CA: Muthén and Muthén

[B24] Schwartz S. H. (1992). Universals in the content and structure of values: theory and empirical tests in 20 countries, in Advances in Experimental Social Psychology, Vol. 25, ed Zanna M. (New York, NY: Academic Press), 1–65

[B25] Schwartz S. H. (2003). A proposal for measuring value orientations across nations, in Questionnaire Development Package of the European Social Survey, Chapter 7. Available online at: www.europeansocialsurvey.org

[B26] Schwartz S. H. (2006). A theory of cultural value orientations: explication and applications. Comp. Sociol. 5, 137–182 10.1163/156913306778667357

[B27] Schwartz S. H. Cieciuch J. Vecchione M. Davidov E. Fischer R. Beierlein C. (2012). Refining the theory of basic individual values. J. Pers. Soc. Psychol. 103, 663–688 10.1037/a0029393 22823292

[B28] Schwartz S. H. Melech G. Lehmann A. Burgess S. Harris M. (2001). Extending the cross-cultural validity of the theory of basic human values with a different method of measurement. J. Cross Cult. Psychol. 32, 519–542 10.1177/0022022101032005001

[B29] Steenkamp J.-B. E. M. Baumgartner H. (1998). Assessing measurement invariance in cross-national consumer research. J. Consum. Res. 25, 78–90 10.1086/209528

[B30] Van de Schoot R. Kluytmans A. Tummers L. Lugtig P. Hox J. Muthén B. O. (2013). Facing off with Scylla and Charybdis: a comparison of scalar, partial, and the novel possibility of approximate measurement invariance. Front. Psychol. 4:770 10.3389/fpsyg.2013.00770 24167495 PMC3806288

[B31] Vandenberg R. J. (2002). Toward a further understanding of and improvement in measurement invariance methods and procedures. Organ. Res. Methods 5, 139–158 10.1177/1094428102005002001

[B32] Vandenberg R. J. Lance C. E. (2000). A review and synthesis of the measurement invariance literature: suggestions, practices, and recommendations for organizational research. Organ. Res. Methods 3, 4–70 10.1177/109442810031002

